# Oligorecurrence Non–small Cell Lung Cancer After Failure of First-Line Chemotherapy: Computed Tomography–Guided ^125^I Seed Implantation vs. Second-Line Chemotherapy

**DOI:** 10.3389/fonc.2020.00470

**Published:** 2020-04-15

**Authors:** Hao Wang, Jian Lu, Xiao-Ting Zheng, Jun-Hao Zha, Wen-Dong Jing, Yong Wang, Guang-Yu Zhu, Chu-Hui Zeng, Lei Chen, Jin-He Guo

**Affiliations:** ^1^Center of Interventional Radiology and Vascular Surgery, Department of Radiology, Zhongda Hospital, Medical School, Southeast University, Nanjing, China; ^2^Center of Oncology, Tianchang City Hospital of Chinese Medicine, Chuzhou, China; ^3^Department of Intervention and Vascular Surgery, The Affiliated Suzhou Hospital of Nanjing Medical University, Suzhou, China

**Keywords:** non–small cell lung cancer, oligometastases, oligorecurrence, ^125^I seed implantation, brachytherapy, chemotherapy

## Abstract

**Purpose:** To compare the efficacy and safety of computed tomography (CT)–guided ^125^I seed implantation with second-line chemotherapy in treatment of oligorecurrence non–small cell lung cancer after failure of first-line chemotherapy.

**Methods:** Data of oligorecurrence non–small cell lung cancer patients after failure of first-line chemotherapy at two institutions were retrospectively reviewed from January 2013 to July 2018. A total of 53 patients who received the treatment of ^125^I seed implantation or second-line chemotherapy were eligible for this study. In group A, 25 patients, 84 lesions, received CT-guided permanent ^125^I seed implantation, whereas in group B, 28 patients, 96 lesions, received second-line chemotherapy. The outcomes were measured in terms of disease control rate, overall survival, quality of life, and complications.

**Results:** The median follow-up period was 13 months (range, 5–42 months). Disease control rate in group A was higher than that in group B (70.8 vs. 42.3%, *P* = 0.042) at 6 months after treatment. The median overall survival was 12.8 months (95% confidence interval, 10.5–15.1 months) in group A and 15.2 months (95% confidence interval, 12.2–18.2 months) in group B, with no significant difference (*P* = 0.847). Since the fourth month, the number of patients in group A with a non-decreasing Karnofsky Performance Scale score was more than that in group B (*P* < 0.05). The incidence of grade 3 or higher complications especially hematologic toxicity in group A was significantly lower than that in group B (*P* < 0.05).

**Conclusion:** Radioactive ^125^I seed implantation is safe and feasible in selected non–small cell lung cancer patients with oligorecurrence after failure of first-line chemotherapy and seems to provide a better long-term quality of life in these patients compared with second-line chemotherapy.

## Introduction

Lung cancer is currently the most common malignant carcinoma with the highest mortality rate in China (1). Approximately 75–80% of the pathological types of lung cancer are non–small cell lung cancer (NSCLC). In NSCLC, 50% of patients who are newly diagnosed are found to have metastases (2). Lung cancer with distant metastases was treated as advanced stage disease and indicated for systemic treatment. Platinum-based chemotherapy has been the standard treatment for metastatic NSCLC. And recently, immunotherapy has become an important treatment strategy for NSCLC (3–6). Despite of recent progress in pharmacotherapy, prognosis of metastatic cancer remains unsatisfactory.

However, not all NSCLC patients with metastasis have a poor prognosis. Hellman and Weichselbaum (7) reported the existence of “oligometastatic state.” These states may be noted at the time of diagnosis (i.e., oligometastasis) or as failure after definitive therapy (i.e., oligorecurrence). Oligometastasis and oligorecurrence refer to patients with metastases limited in number and location, which represents an intermediate state between locally confined and widely metastatic cancer. There is currently no clear definition of oligometastatic disease. Most clinical trials and clinicians accept a definition of fewer than five metastatic lesions (8–11). Many studies indicated that patients with oligometastases might benefit from local treatment, such as surgical resection, radiotherapy, and radiofrequency ablation (12–14). Stereotactic body radiotherapy (SBRT) is a specialized form of radiotherapy, which is characterized by higher doses of radiation, shorter time period, and more precise targeting system. Stereotactic body radiotherapy has become a preferred treatment strategy for oligometastatic disease (13, 15).

Radioactive ^125^I seed (RIS) brachytherapy recently has been a standard treatment in early low- to intermediate-risk prostate cancer (16, 17). It is a type of low-dose-rate brachytherapy, which could deliver high radiation dose to target tumor while safely sparing the adjacent normal tissue. Previous studies indicated that ^125^I brachytherapy is a feasible salvage treatment method for many cancers, including prostate cancer, lung cancer, pancreatic cancer, and head and neck cancer (18). To a certain extent, RIS implantation brachytherapy is similar to a single dose of stereotactic ablative radiotherapy and can also be called “stereotactic ablative brachytherapy” (16). However, whether RIS implantation could provide benefits in terms of local control and survival to oligorecurrence NSCLC patients after failure of first-line chemotherapy remains unclear.

The purpose of this study was to compare the efficacy and safety of computed tomography (CT)–guided ^125^I seed implantation with second-line chemotherapy in treatment of oligorecurrence NSCLC after failure of first-line chemotherapy.

## Materials and Methods

This study was approved by the institutional review board. From January 2013 to July 2018, data of consecutive case of stage IV NSCLC who experienced failure of first-line chemotherapy were queried from electronic medical record. Those patients did not have history of polymetastatic disease before oligometastatic NSCLC diagnosis, while they had history of oligometastatic disease. And oligometastatic disease is diagnosed within 6 months after diagnosis of the primary tumor. Those patients had newly developed metastases after receiving first-line chemotherapy (paclitaxel + cisplatin, gemcitabine + cisplatin) four to six cycles. According to the characterization and classification of oligometastatic disease consensus recommendation, those patients were defined as repeat oligoprogression (19). Those patients who underwent CT-guided percutaneous RIS implantation (group A) or second-line chemotherapy (group B) were included in this study. Patients who received RIS implantation signed an informed consent form. It is important to point out that both primary tumor and metastatic sites received RIS implantation. The inclusion criteria were (1) histologically proven NSCLC, (2) negative driver mutation, (3) intolerant to surgical resection and external beam radiotherapy, (4) fewer than 5 metastatic nodules, (5) absence of severe coagulation dysfunction, and (6) Karnofsky Performance Scale (KPS) score ≥70. The exclusion criteria were as follows: (1) with other types of primary tumors except NSCLC and (2) with incomplete data. After removing patients who were lost to follow-up or with missing data, a total of 53 patients were finally eligible for this study.

### Treatment

#### ^125^I Radioactive Seed Implantation

##### Instruments

Brachytherapy treatment planning system (Qilin Co., Ltd., Peking, China) was applied to calculate ^125^I seed dose distribution, according to the American Association of Physicists in Medicine TG43 brachytherapy formalism (20). The parameters of ^125^I seed (XinKe Pharmaceutical Ltd., Shanghai, China) were 0.8 mm in diameter, 4.5 mm in length, and 0.05 mm in thickness of the wall of the titanium capsule. The gamma rays delivered by ^125^I (5% of 35 keV, 95% of 28 keV) was with a half-life of 59.6 days, penetration of 17 mm, incipient rate of 7 cGy/h, and activities of 0.5–0.8 mCi. Eighteen-gauge implantation needles and turntable implantation gun (XinKe Pharmaceutical Ltd.) were applied for the RIS implantation.

##### CT-guided implantation protocol

**Figure 1:**
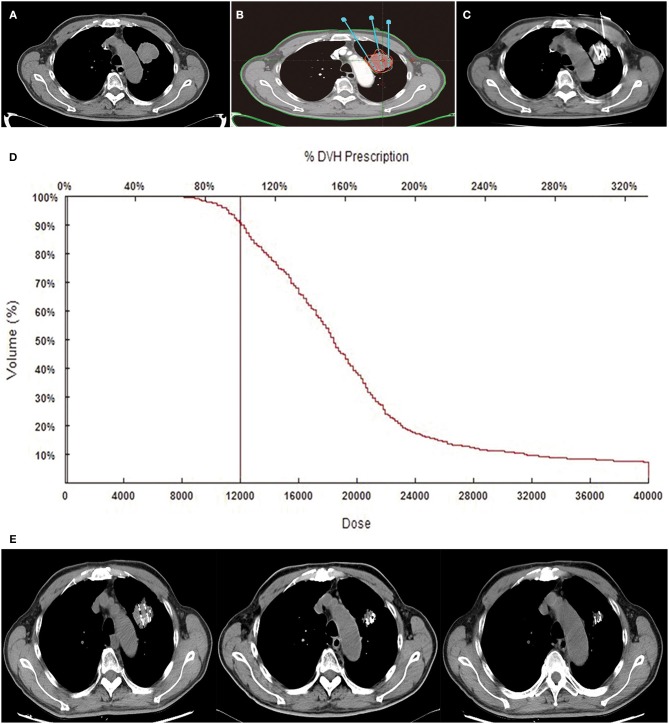
Tumor, treatments, and follow-up CT. An 81-year-old male patient with NSCLC received RIS implantation. Preoperative CT images showed the oligometastatic lesion was located in the upper lobe of the left lung **(A)**. Isodose curves and planning target volume plotted by TPS. The red line shows the planning target volume, and the green line shows the 120-Gy isodose curves **(B)**. A total of 30 ^125^I seeds were implanted into the tumor under CT guidance, and the radioactivity of the seed was 0.6 mCi **(C)**. Dose–volume histograms (DVH). The prescription dose is 120 Gy during planning. A total of 90% of the tumor target received 120.0 Gy, and 95.4% of the tumor received 100% of the prescribed dose **(D)**. Gradually, the mass shrunk at the second, fourth, and sixth months on the following CT images **(E)**.

All patients received CT (Siemens, Munich, Germany) scan with 5-mm slice thickness and spacing 2–3 days before RIS implantation (Figure 1A). Then CT images were uploaded into a treatment planning system. Radiation physicians outlined the gross tumor volume (GTV) on each transverse image and determined the prescription dose [according to international standards for prostate cancer and expert consensus on RIS brachytherapy (18, 21)]. And the total number and activity of seed to be implanted were calculated via the modified level formula (22) (Figure 1B). The dosimetric goal was that the dose received by 90% of the GTV should reach the prescription dose as much as possible, and the doses delivered to the adjacent normal organs were as low as possible (16).

As radiation sources, ^125^I seeds were implanted into primary and metastatic lesions under CT guidance, at a spacing of 1 cm. Prescription dose was 100–140 Gy. Activity of ^125^I seeds was 0.4–0.8 mCi. All the RIS implantation procedures were performed by interventional radiologists in a standard CT room under local anesthesia. The space between the adjacent implantation needles was ~1 cm each. Repeated CT allowed adjustment of depth and angle of needles to avoid adjacent vessels and organs. Seeds were released every 0.5–1.0 cm apart upon withdrawing the needles (Figure 1C). After RIS implantation, patients were observed in the interventional radiology wards for 1–2 days.

##### Postimplantation dosimetry

After the completion of RIS implantation, immediately CT scan should be performed to validate the actual post-operative distribution of RIS. Then CT images would be uploaded into TPS software, a redundancy check was performed to prevent seed duplication. Dosimetric parameters, including D90 and V100, were used to evaluate the dosimetry. D90 represents the dose delivered to the 90% of GTV, and V100 means the percentage of GTV receiving 100% of prescription dose. Actual isodose distributions for each slice and dose volume histograms for the target were generated (Figures 1D,E).

##### Second-line chemotherapies

The chemotherapy regimen for squamous cell carcinoma patients was docetaxel 75 mg/m^2^ per 3 weeks, and that for non-squamous cell carcinoma was pemetrexed 500 mg/m^2^ per 3 weeks. The drug dose was modified on the basis of blood cell counts and renal function on the day of therapy. During chemotherapy, routine blood test and coagulation function plus liver and kidney function tests were performed.

##### Follow-up

Patients who underwent ^125^I radioactive seed implantation were closely observed for their vital signs within 24 h, with electrocardiogram monitoring performed and all post-operative complications recorded in detail. Follow-up of all patients was carried out at an interval of 2 months, including disease control rate (DCR), quality of life (QOL), complications, and death time. Dynamic enhanced CT, brain magnetic resonance, and laboratory test (routine blood test and coagulation function plus liver and kidney function) were performed routinely.

##### Outcomes and definitions

The primary endpoint is DCR. In this study, DCR refers to disease control of both primary tumor and metastatic sites. It is overall response assessment, which is a result of the combined assessment of current lesions and new lesions. Tumor response was evaluated according to the Response Evaluation Criteria in Solid Tumors (RECIST 1.0): complete response (CR): all target lesions disappear, confirmed at 4 weeks; partial response (PR): baseline lesion total diameter reduction ≥30%, confirmed at 4 weeks; stable disease (SD): between PR and PD; progression disease (PD): total length of lesion increased ≥20% or new lesions. Disease control rate was calculated as (CR + PR +S D)/total number of patients × 100%. The result of tumor response was confirmed by two to three senior radiologists. Quality of life was evaluated according to KPS score. The improvement of QOL was defined as an increase of at least 10 points of KPS after treatment, whereas the worsening of QOL referred to a decrease of 10 points or more, and the stability of QOL meant that the fluctuation of KPS was <10 points. The overall survival (OS) was defined as the time interval from initial treatment to death or the last follow-up. The Common Terminology Criteria for Adverse Events 4.0 was used to assess treatment-related adverse effects.

##### Statistical analysis

Numerical data with normal distribution were expressed as mean ± SD, whereas data with non-normal distribution were expressed as median (interquartile range). Continuous variables were compared using the *t*-test or Mann-Whitney *U* test for variables with a normal or non-normal distribution. Categorical variables were compared using the χ^2^ test or Fisher exact test. Overall survival time analyses were performed with the Kaplan–Meier method and log-rank test. *P* < 0.05 was considered statistically significant. All data analyses were performed using SPSS 18.0 software (IBM, Armonk, NY, USA).

## Results

### Patient Characteristics

In group A, 25 patients received CT-guided percutaneous RIS implantation. As shown in Table 1, 17 male and 8 female patients, with a median age of 68 years (range, 38–84 years), were evaluated. Fifteen cases were adenocarcinomas, and 10 were squamous cell carcinomas. Oligometastatic sites were located in lung (*n* = 9), adrenal gland (*n* = 7), liver (*n* = 5), and lymph nodes (*n* = 4). Metastatic lymph nodes were located in supraclavicular and mediastinal lymph nodes. In group A, 25 patients, 84 lesions [lung (31), adrenal gland (24), liver (16), and lymph nodes (13)], received CT-guided ^125^I seed implantation. In group B, 28 patients, 96 lesions [lung (36), adrenal gland (23), liver (21), and lymph nodes (16)], underwent second-line chemotherapy with docetaxel or pemetrexed. The baseline characteristics of these patients are summarized in Table 1.

**Table 1:** Patient characteristics.

	**Group A *n* = 25**	**Group B *n* = 28**	***P***
Age (years)Range	38–84	45–82	0.941
Mean	69	69	
Gender			0.776
Male	17	18	
Female	8	10	
Histology			0.958
Squamous carcinoma	10	11	
Adenocarcinoma	15	17	
Number of lesions	84	96	0.079
Oligometastatic site			0.069
lung	9	10	
Adrenal gland	7	5	
Liver	5	7	
Lymph nodes	4	6	
Tumor size ≤ 3 cm	12	16	0.506
>3 cm	13	12	
KPS			0.158
70	3	2	
80	4	5	
90	10	11	
100	8	8	

*KPS, Karnofsky Performance Scale score; SD, standard deviation*.

### Disease Control Rate

At the second, fourth, and sixth months after treatment, DCR in group A vs. group B was 76.0 vs. 75.0%, 72.0 vs. 55.6%, and 70.8 vs. 42.3% (*p2* = 0.933, *p*4 = 0.219, *p*6 = 0.042), respectively (Table 2). Disease control rate of group A was statistically higher than that of group B at the sixth month.

**Table 2:** Disease control in two groups.

	**CR**	**PR**	**SD**	**PD**	**DCR (%)**	**Total**	P^*^
**Group A**							
2nd	1	8	10	6	76.0	25	0.933
4th	1	8	9	7	72.0	25	0.219
6th	1	6	10	7	70.8	24	0.042
**Group B**							
2nd	0	5	16	7	75.0	28	
4th	0	5	10	12	55.6	27	
6th	0	2	9	15	42.3	26	

*CR, complete response; PR, partial response; SD, stable disease; PD, progressive disease; DCR, disease control rate*.

* *P-value was the comparison result of two groups of DCR at second, fourth, and sixth months, respectively*.

### Quality of Life

At the second, fourth, and sixth months after treatment, the numbers of patients with non-decreasing KPS (increase and stable) in group A vs. group B were 22 (88.0%) vs. 20 (71.4%), 18 (72.0%) vs. 12 (48.1%), and 15 (62.5%) vs. 8 (30.8%) (*p2* = 0.138, *p*4 = 0.044, *p*6 = 0.025). No significant differences were observed between two groups at the second month after the treatment, whereas at the fourth and sixth follow-up, the difference in QOL between two groups turned out to be significant (Table 3).

**Table 3:** The quality of life of patient in two groups.

	**KPS**				
	**Increase**	**Stability**	**Decrease**	**No decrease/total (%)**	P^*^
**Group A**					
2nd	4	18	3	88.0	0.138
4th	3	15	7	72.0	0.044
6th	3	12	9	62.5	0.025
**Group B**					
2nd	1	19	8	71.4	
4th	2	10	15	48.1	
6th	1	7	18	30.8	

* *P-value was the comparison result of two groups on KPS at second, fourth, and sixth months, respectively*.

### Complication

Treatment-related deaths were not observed in the two groups. The incidence rate of nausea/vomiting, neutropenia, and fatigue in group A was statistically lower than that in group B (Table 4). In group B, 5 patients (17.9%) had grade ≥3 neutropenia, and 2 (7.1%) had grade ≥3 nausea/vomiting. In group A, CT images showed four patients had pneumothorax, and two of them had pulmonary tissue compression of more than 30%, which needed catheter drainage, and recovered in 2–3 days. Since pulmonary tissue compression was <30% and no significant symptoms appeared, the other two pneumothorax patients did not receive any clinical treatment and recovered spontaneously in a week. In group A, one patient manifested self-limited hemoptysis for ~20 mL, and another one presented bloody sputum. The symptoms disappeared soon without any treatment.

**Table 4:** Treatment-related complications in two groups.

**Complications**	**Group A** ***n*** **= 25**				**Group B** ***n*** **= 28**				P^*^
**Grade**	**1**	**2**	**≥3**	**Total (%)**	**1**	**2**	**≥3**	**Total (%)**	
Neutropenia	2	0	0	8.0	2	2	5	32.1	0.031
Nausea/vomiting	1	0	0	4.0	4	2	2	28.6	0.044
Fatigue	2	0	0	8.0	6	3	0	32.1	0.031
Hemoptysis	2	0	0	8.0	0	0	0	0	
Pneumothorax	2	0	2	16.0	0	0	0	0	

* *P-value was the comparison result of two groups on treatment-related complications*.

### Overall Survival

**Figure 2:**
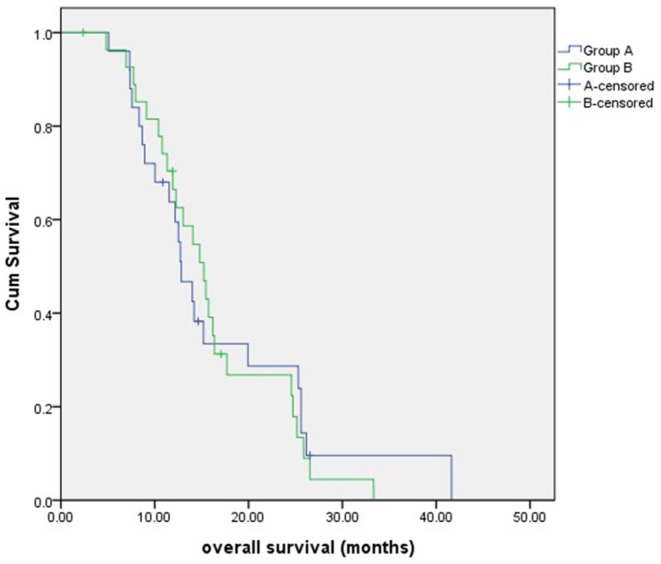
The overall survival of patients in two groups. There was no significant difference between groups A and B.

The median follow-up time was 13 months (range, 5–42 months). By the end of the follow-up period, 22 patients died in group A, and 25 patients died in group B. The 1-year OS and 2-year OS for group A vs. group B were 59.5vs. 62.6% and 23.9 vs. 22.3%, respectively. The median OS was 12.8 months (95% confidence interval, 10.5–15.1 months) in group A and 15.2 months (95% confidence interval, 12.2–18.2 months) in group B, with no significant difference (*P* = 0.847) (Figure 2).

## Discussion

The present study indicates that CT-guided RIS implantation is safe and feasible in NSCLC with oligorecurrence after failure of first-line chemotherapy and seems to provide a better long-term QOL in these patients compared with second-line chemotherapy.

At present, many patients with oligometastatic cancer receive systemic therapy, while their physical strength is usually impaired. In most situations, long-term survival could not be always achievable; thus, in order to allow them to live their remaining lives with a better QOL, less invasive local treatment strategies are desirable (13). Stereotactic body radiotherapy can provide a high level of local control with less associated adverse events, which has become one of the preferred modalities for local ablation of oligometastatic disease. ^125^I brachytherapy has been applied for primary treatment of cancer for decades and can also be called “stereotactic ablative brachytherapy.” ^125^I brachytherapy and SBRT have some similarities as a highly precise local therapy. ^125^I brachytherapy can deliver high radiation dose, because of accumulated radiation dose delivered continuously by ^125^I seeds and localized in the target tumor. Therefore, the adjacent normal tissues could be spared (16). However, a few complications associated with needle puncture should be noticed.

Recently, RIS implantation brachytherapy has been successfully used to treat diverse kinds of malignant tumors (17, 23–26). A large quantity of studies on RIS implantation for NSCLC have been reported with promising results. A small sample study suggests that ^125^I seed implantation for lung cancer patients was safe, and no complications were observed (27). A meta-analysis on 1,188 cases from 15 clinical studies suggests that ^125^I seed implantation combined with chemotherapy could improve the efficacy without increasing the incidence of adverse effects for advanced NSCLC (28).

In group A, the total incidence rate of treatment-related complications was lower than that in group B. Although pemetrexed and docetaxel are considered the standard second-line chemotherapy regimens (29), drug toxicity cannot be ignored. In a randomized controlled trial (30), patients who received second-line chemotherapy with docetaxel presented grade ≥3 adverse effects, including neutropenia (24%), leukopenia (11%), and febrile neutrophils reduction (7%), and those who cannot tolerate drug toxicity had to cease the treatment.

In this study, the incidence of myelosuppression in group A was significantly lower than that in group B (8.0 vs. 32.1%, *P* = 0.031). Actually, because the bone marrow is highly radiosensitive, external beam radiotherapy can cause suppression on bone marrow system (31). A study shows that for advanced NSCLC patients who received external beam radiotherapy, the incidence of grade 2 myelosuppression was 16.7% (32). In our study, the incidence of myelosuppression was 8.0%, which was lower than external beam radiotherapy caused. We speculated that it may be attributed to the short irradiation distance and low dose rate of ^125^I radioactive seeds, which reduce irradiation damage on hematopoietic cells in bone marrow. In addition, the toxicity induced by ^125^I seed, such as gastrointestinal reaction, radiation esophagitis, radiation pneumonia, and radiation dermatitis, is rarely observed, which is similar to previous studies (28).

Patients who received percutaneous RIS implantation have a high relative risk of pneumothorax. A study reports that seven patients with NSCLC were treated with percutaneous ^103^Pd or ^125^ I seed implantation, and two patients developed pneumothorax (33). In this study, four patients (16.0%) had pneumothorax in group A. Usually, the occurrence of pneumothorax may be related to tumor location, lung function, puncture needle diameter, rib obstruction, and respiratory movement. In this study, four patients all received repeated punctures, owing to rib blockage and respiratory movement.

Radioactive ^125^I seed implantation has advantages of relieving tumor-related symptoms and improving QOL (34). A study shows that, for advanced NSCLC patients after failure of first-line chemotherapy, ^125^I seed implantation could improve DCR and relieve chest pain, cough, and shortness of breath without increasing the incidence of treatment-related adverse effects (35). In this study, the difference in QOL between two groups manifested significant since the fourth-month follow-up. In the long run, ^125^I brachytherapy seems to provide a better long-term QOL than second-line chemotherapy. Computed tomography–guided ^125^I implantation is a minimally invasive treatment with a low incidence of complications, which may help improve the long-term QOL of patients. Meanwhile, we noted that the difference in QOL between two groups manifested significant since the fourth-month follow-up. And the accumulated dose that tumor absorbed increases as irradiation time moves along. Therefore, RIS implantation gradually showed an advantage over disease control since the fourth month.

Interestingly, similar phenomena were observed in terms of DCR. ^125^I brachytherapy is a type of low-dose-rate therapy because the dose delivered by RIS implanted permanently in place is <1 Gy per h. Meanwhile, ^125^I brachytherapy can also be treated as high-dose-rate therapies, in which the radiation is delivered over several courses (36, 37). The accumulated dose of tumor and efficacy increase as irradiation time moves along. Radioactive ^125^I seed has a half-life of nearly 2 months, meaning half of the energy is released in 2 months. The feature of ^125^I radioactive seed may be associated with the above result.

There are some limitations to our research. The sample size was small, and the duration of follow-up was short. Also, the retrospective aspect of this study could not provide sufficient evidence. Therefore, multicenter randomized controlled trails should be carried out in the future. One limitation that has to be pointed out is that RIS implantation is considered a local treatment, whereas second-line chemotherapy is a systemic therapy. However, when it comes to clinical significance, a treatment that is more effective for patients who failed first-line chemotherapy is the one that should win the most concern.

In summary, the present study indicates that RIS implantation is safe and feasible in NSCLC with oligorecurrence after failure of first-line chemotherapy and seems to provide a better long-term QOL in these patients compared with second-line chemotherapy.

## Data Availability Statement

All datasets generated for this study are included in the article/supplementary material.

## Ethics Statement

Ethical review and approval was not required for the study on human participants in accordance with the local legislation and institutional requirements. The patients/participants provided their written informed consent to participate in this study. Written informed consent was obtained from the individual(s) for the publication of any potentially identifiable images or data included in this article.

## Author Contributions

HW, JL, and J-HG contributed to study concept and design. HW, X-TZ, and W-DJ contributed to acquisition of data. HW, J-HZ, C-HZ, and JL contributed to drafting of the manuscript. J-HG, YW, LC, and G-YZ contributed to analysis and interpretation of data. HW contributed to statistical analysis. J-HG and G-YZ supervised and oversaw the study. The corresponding authors had full access to all of the data and take full responsibility for the veracity of the data and the statistical analyses. All authors contributed to review and critical revision of the manuscript and approved the final version of the manuscript.

### Conflict of Interest

The authors declare that the research was conducted in the absence of any commercial or financial relationships that could be construed as a potential conflict of interest.
